# *Elizabethkingia miricola* as an opportunistic oral pathogen associated with superinfectious complications in humoral immunodeficiency: a case report

**DOI:** 10.1186/s12879-017-2886-7

**Published:** 2017-12-12

**Authors:** Przemysław Zdziarski, Mariola Paściak, Klaudia Rogala, Agnieszka Korzeniowska-Kowal, Andrzej Gamian

**Affiliations:** 1 Department of Clinical Immunology, Lower Silesian Center for Cellular Transplantation, PO Box 1818, 50-385 Wrocław-46, Poland; 2 Hirszfeld Institute of Immunology and Experimental Therapy, Polish Academy of Sciences, Rudolfa Weigla 12, 53-114 Wroclaw, Poland

**Keywords:** *Elizabethkingia miricola*, Superinfection, Portal of entry, Periodontitis, Natural history of disease, MALDI-TOF Biotyper

## Abstract

**Background:**

*Elizabethkingia miricola* is a rare Gram-negative bacterium found in water and clinical specimens. Typical culturing methods often misidentify *Elizabethkingia* spp. as *Flavobacterium* or *Chryseobacterium*. Although diagnosis is based on culturing samples taken from sterile sites, such as blood, a proper identification of this bacterium requires an expertise that goes beyond the capabilities of a typical clinical laboratory.

**Case presentation:**

A 35-year-old woman diagnosed with common variable immunodeficiency was admitted to our center. Previous treatment with antibiotics (amoxicillin plus clavulanate, first and third generation of cephalosporins, macrolides) and systemic corticosteroids (up to 120 mg/day of prednisolone) failed to arrest the spread of inflammation. Gingival recession was observed in her oral cavity, resulting in an apparent lengthening of her teeth. In addition to typical commensal bacteria, including streptococci and neisseriae, strains of *Rothia mucilaginosa* and *Elizabethkingia miricola* were identified upon a detailed microbiological examination using a MALDI-TOF MS Biotyper system. The presence of the latter strain correlated with severe periodontitis, lack of IgA in her saliva and serum, a very low IgG concentration (< 50 mg/dl), IgM-paraproteinemia, decreases in C3a and C5a and microvascular abnormality. High-dose immunoglobulin (to maintain IgG > 500 mg/dl) and targeted levofloxacin treatment resulted in immune system reconstitution, oral healing, and eradication of the *Elizabethkingia* infection.

**Conclusions:**

*E. miricola* rarely causes disease in healthy individuals. However, the overgrowth of commensal bacteria, lack of IgG/IgA, microvasculopathy and complement cascade activation in patients with humoral immunodeficiency may facilitate *Elizabethkingia* invasion. Overuse of antibiotics, particularly beta-lactams, may cause mucosal colonization by *E. miricola,* followed by its multiplication combined with periodontitis that prompts bacterial translocation. MALDI-TOF Biotyper analysis may become a method of choice for identification of *Elizabethkingia* infections.

**Electronic supplementary material:**

The online version of this article (10.1186/s12879-017-2886-7) contains supplementary material, which is available to authorized users.

## Background

The genus *Elizabethkingia* contains three medically important species: *E. meningoseptica*, *E. anophelis* and *E. miricola*. Despite the clinical importance of *E. miricola* infections, this bacterium has not been characterized epidemiologically, as its route of transmission and portal of entry remain poorly understood. This bacterium usually induces pneumonia and lower respiratory tract infections, with the latter consisting of the formation of pulmonary nodules followed by sepsis [1], lung abscess and pleural effusion [2], or severe nosocomial pneumonia [3]. However, the pathogenesis of established infections has not been clarified. Most clinical isolates of the genus *Elizabethkingia* come from patients infected by *E. meningoseptica* and having severe forms of infection, such as meningitis, sepsis, and pneumonia [4]. Moreover, most clinical isolates have been obtained from patients in Asia and Africa [5], areas in which *E. anophelis* and *E. meningoseptica* are highly prevalent in the gut of *Anopheles* mosquitoes [6, 7], with horizontal transfer having been observed within a colony of *Anopheles gambiae* [8, 9]. However, vector-borne transmission has not been reported for *E. miricola* and, although cases of *E. miricola* infections have been reported, the epidemiology of this species remains unclear [1]. *E. miricola* has also been isolated from synovial fluid [10] and urine samples [11] of European patients; these sites are normally sterile sites, with the mode of entering a susceptible host remaining unclear. The presence of *E. miricola* in urine suggests septicemia, which can be fatal if not treated early with appropriate antibiotics. In addition, *E. miricola* has been isolated from the blood of a patient with acute alcoholic pancreatitis [12].

Most clinical laboratories are unable to identify *Elizabethkingia* at the species level. New techniques, such as matrix-assisted laser desorption/ionization time-of-flight (MALDI-TOF) mass spectrometry, have been utilized recently for microbiological typing [10]. In the near future and following the construction of appropriate reference spectrum databases, MALDI-TOF mass spectrometry may become a method of choice for identifying pathogens [5, 13].

Little is known about the predisposing factors, preclinical phase, web of causation, pathologic evidence and period of communicability of *E. miricola* infection. Host-pathogen interactions in the colonization phase may be crucial for subsequent invasion at the portal of entry. Understanding these steps can help in developing preemptive therapies and assessing risk factors in patients with primary immunodeficiency.

This case report describes a patient who experienced a primary *E. miricola* infection and the complex diagnostic process and successful preemptive therapy in this patient. To our knowledge, this study describes the first isolation of *E. miricola* from a non-sterile specimen of a patient with a well-defined primary immunodeficiency before she developed serious and systemic complications. Written consent to publish this report was obtained from the patient.

## Case presentation

### Medical history of the patient

A 35-year-old woman was recently admitted to our center and diagnosed with common variable immunodeficiency and infectious complications (streptococcal pharyngitis and recurrent pneumonia, predominantly pneumococcal). Previously, following examination by a general practitioner, she received empirical antibacterial therapy, consisting of high dose amoxicillin (1000 mg t.i.d.) plus clavulanate (200 mg t.i.d.), followed by cefadroxil (500 mg b.i.d.), ceftriaxone (1000 mg q24h), and azithromycin (500 mg q24h). Her medical history included recurrent, multiple ulcers in the oral cavity (a case report timeline is shown in the Additional file 1: Figure. S1). The patient had periodontitis with gingival recession and was spitting out blood after brushing her teeth (Additional file 2: Figure. S2). Before hospitalization, she experienced mucosal pyogenic granulomas and easy bleeding, without coagulation factor deficiency. Nonsurgical cleaning below the gum line was ineffective. At the time of admission, cold agglutinin and Raynaud phenomenon were observed (Additional file 3: Table S1). Progressive microvascular complications of hyperviscosity with life-threatening symptoms were observed, including progressive weakness and focal neurologic signs with paresthesia, headaches, and positive Kernig’s and Brudzinski’s signs (meningismus). Examination of her cerebrospinal fluid and magnetic resonance imaging showed no abnormalities. Following treatment with crystalloids, no rouleau formation was observed.

Laboratory data showed a normal B cell count, but undetectable IgG (< 50 mg/dl) and IgA (< 1 mg/dl) (Additional file 3: Table S1). Behcet disease, X-linked CD40/CD40L mutations and secondary agammaglobulinemia were excluded according to ESID criteria for common variable immunodeficiency (CVID) [14]. Her initial IgM concentration was very high (1789 mg/l) and increased to 2235 mg/l, with hyperviscosity-like syndrome and neuropathy. The presence of < 1% lymphoplasmacytic cells in her bone marrow and the absence of monoclonal protein excluded lymphoma and Wiskott-Aldrich syndrome, respectively. Because her severe humoral immunodeficiency and meningismus suggested a bacterial origin of her symptoms, a strict microbiological assessment was performed.

### Bacterial culture and identification

Bacteria were cultivated on brain heart infusion (BHI) agar, thioglycolate-soy agar, nutrient agar and blood agar at 37 °C for 24–48 h under aerobic and anaerobic conditions (GasPakTM). Single cell colonies were processed using an extraction protocol [13] and bacterial strains were identified by MALDI-TOF MS. All analyses were performed with an Ultraflextreme mass spectrometer (Bruker Daltonics, Germany) using the Biotyper 3.1 software and database containing 4613 entries [13].

Microorganisms taken from the oral cavity with bacterial swabs were cultivated for 24 h under aerobic conditions. Gram-positive strains were isolated, with the dominant strain being the potentially pathogenic *Staphylococcus aureus*. Oral streptococci were also isolated, including *Streptococcus salivarius*, *S. sanguinis*, *S. gordonii* and *S. oralis*, all of which were considered commensal, non-periodontopathogenic bacteria [15]. Apart from streptococci, *Rothia mucilaginosa* and *Neisseria* spp., we found a rare Gram-negative species, *Elizabethkingia miricola* (Table 1). Its identification with MALDI-TOF MS was later confirmed by 16S rRNA analysis.

**Table 1:** Clinical strains isolated from the patient and identified by MALDI-TOF MS Biotyper system

Strain	Conditions	Score value^a^
*Staphylococcus aureus*	Aerobic	2.346
*Rothia mucilaginosa*	Aerobic	2.443
*Neisseria mucosa*	Aerobic	2.29
*Neisseria cinerea*	Aerobic	2.2
*Neisseria flavescens*	Aerobic	2.256
*Elizabethkingia miricola*	Aerobic	2.18
*Streptococcus salivarius*	Aerobic	2.171
*Streptococcus sanguinis*	Aerobic	2.276
*Streptococcus gordonii*	Aerobic	1.771
*Veillonella parvula*	Anaerobic	2.043
*Streptococcus oralis*	Anaerobic	2.194

^a^Score values determined by MALDI Biotyper: < 1.7 – identification not reliable, 1.7–2.0 – probable genus identification, 2.0–2.3 – secure genus identification and probable species identification, > 2.3 – highly probable species identification

Chromosomal DNA was isolated [16], and a ~500-bp fragment of the 16S rRNA gene was amplified using the universal primers, 16S_Start 27F (5’-AGAGTTTGATCMTGGCTCAG-3′) and 16S_Stop 519R (5’-GWATTACCGCGGCKGCTG-3′). The products were sequenced by Sanger’s method, and the sequences were aligned using SeqAssem ver.07/2008. Homology with known sequences was searched using the EzTaxon database (http://www.ezbiocloud.net). The 522 bp sequence of the clinical isolate showed the highest homology (99.37%) with the 16S rRNA gene sequence of *Elizabethkingia miricola* GTC862^T^, exhibiting three different nucleotides in a 475 bp fragment. The strain isolated has been deposited in the Polish Collection of Microorganisms (*E. miricola* PCM 2858).

### Antimicrobial susceptibility test

Antimicrobial susceptibility of the clinical isolate was tested by Vitek 2 (bioMérieux, Marcy l’Etoile, France) and by the disc diffusion method. The isolate *E. miricola* PCM 2858 was susceptible to ciprofloxacin (MIC 0.25 mg/L), levofloxacin (MIC 0.25 mg/L), and trimethoprim-sulfamethoxazole, but resistant to all the other drugs tested (Table 2).

**Table 2:** Results of antimicrobial susceptibility testing for clinical isolate *E. miricola*

Antimicrobial^a^	MIC (mg/L)	MIC breakpoint (mg/L)		Interpretation
		Susceptible ≤	Resistant >	
Cefepime	≥32	4	8	Resistant
Piperacillin	≥128	4	16	Resistant
Piperacillin/Tazobactam	≥128	4	16	Resistant
Ticarcillin/Clavulanic acid	≥128	8	16	Resistant
Ciprofloxacin	≤0.25	0.5	1	Susceptible
Levofloxacin	0.25	1	2	Susceptible
Meropenem	≥16	2	8	Resistant
Gentamicin	8	4	4	Resistant
Amikacin	≥64	8	16	Resistant
Tobramycin	≥16	4	4	Resistant
Colistin	≥16	4	4	Resistant
Antimicrobial^b^	Inhibition diameter (mm)	Zone diameter breakpoint (mm)		Interpretation
		Susceptible ≥	Resistant <	
Ceftazidime	6	17	17	Resistant
Cefepime	12	19	19	Resistant
Trimethoprim/Sulfamethoxazole	24	16	16	Susceptible
Amikacin	13	18	15	Resistant
Gentamicin	14	15	15	Resistant
Imipenem	6	20	17	Resistant
Meropenem	6	24	18	Resistant
Levofloxacin	27	20	17	Susceptible
Ciprofloxacin	28	25	22	Susceptible

^a^Antimicrobial susceptibility testing using Vitek 2 (bioMérieux, Marcy l’Etoile, France), according to the European Committee on Antimicrobial Susceptibility Testing (EUCAST) guidelines 2016 (http://www.eucast.org). For interpretation, non–species-related (pharmacokinetics/ pharmacodynamics) breakpoints were used except for gentamicin, amikacin, tobramycin and colistin, in which *Pseudomonas* sp. breakpoints were used

^b^Antimicrobial susceptibility testing by the disc diffusion method according to 2016 EUCAST guidelines (http://www.eucast.org). For interpretation, non–species-related (pharmacokinetics/ pharmacodynamics) breakpoints were used except for trimethoprim/ sulfamethoxazole, in which *Stenotrophomonas maltophilia* breakpoints were used, and for gentamicin, amikacin, tobramycin, ciprofloxacin and levofloxacin, in which *Pseudomonas* sp. breakpoints were used

### Treatment

Following the diagnosis of CVID and the identification of the bacteria, the patient was started on levofloxacin (750 mg/day for 21 days) and IgG replacement therapy with intravenous immunoglobulins (IVIG; 0.4 g per kg body weight every week for 3 weeks to achieve a trough IgG level, measured before the next infusion, of at least 500 mg/dl). Complete eradication of *E. miricola* and complete healing of the mucosal ulcerations were observed 48 days after initial IVIG replacement therapy, when a stable IgG level of 500–800 mg/dl was first achieved.

## Discussion and conclusions

Periodontitis is caused by bacteria that adhere to and grow on the surfaces of teeth. In periodontal disease, host protection first occurs at the level of innate gingival epithelial immunity. This is followed by an adaptive immune response against these microorganisms; this response, however, is impaired in patients lacking specific IgG/IgA. Periodontal tissue pathology results when matrix metalloproteinases are released from bacteria or neutrophils [17]. Although non-specific immune response against these microorganisms has been observed, we found that topical and systemic steroid treatments were ineffective, whereas levofloxacin and IVIG were successful. These findings suggested that humoral immunodeficiency, complement activation and modification of mucosal biofilm by antibiotics induced the overgrowth of commensal bacteria (e.g., *Rothia* spp.) and abnormal opportunistic infection by *Elizabethkingia* in our patient (Additional file 4: Figure. S3).

*Elizabethkingia* spp. are considered to be multi-drug resistant. The first described strain of *E. miricola* was isolated from severely immunocompromised patients and thought to cause nosocomial pneumonia [1]. This strain was susceptible to ciprofloxacin (MIC ≤1 μg/ml) and levofloxacin (MIC 2 μg/ml) but resistant to all other antibiotics tested, including aminoglycosides; first, second and third-generation cephalosporins; carbapenems; trimethoprim/sulfamethoxazole; and colistin. Our isolate of *E. miricola* showed an antimicrobial susceptibility pattern similar to that of *E. miricola* isolate HvH-WGS333 [10]. Unlike *E. miricola* EM_CHUV, however, our strain was resistant to amikacin and gentamicin [3]. A study of the resistome of *E. miricola* found 40 antibiotic resistance genes, many of which are responsible for resistance to β-lactams [3]. Interestingly, *E. miricola* was found to produce metallo-β-lactamases [11].

Aerobic/anaerobic, Gram-positive and Gram-negative bacteria have been found to coexist frequently in biofilms. Similarly, in the patient described here *E. miricola* coexisted with many other bacterial species, suggesting that superinfection was a source of *Elizabethkingia* colonization, as the core microbiome of the oral cavity does not contain *Elizabethkingia* spp. [17]. Previous results, although without strict epidemiological data [4, 5, 11], support the concept of superinfection. Superinfection is generally defined as a second infection superimposed on an earlier infection, with the superinfecting pathogen generally resistant to the agent used to treat the initial infection (e.g., *Clostridium difficile*). The empirical regimen used in our patient, including the overuse of wide-spectrum antibiotics, such as amoxicillin plus clavulanate, first and third generation cephalosporins, and macrolides, may have been responsible, at least in part, for the superinfection and the antibiotic resistance of Gram-negative bacteria. Interspecies competitions among *Elizabethkingia* and streptococci, as well as the neisserial biofilm, may be affected by the overuse of beta-lactam antibiotics [18].

Infection begins when a foreign organism successfully enters the body through a portal of entry. One such portal is the oral cavity, which experiences a high influx of bacteria that produce matrix metalloproteinases and are responsible for gingival recession and periodontitis. Persistent bleeding after minor procedures, such as spitting out blood after tooth brushing, likely promotes bacterial translocation. Severe humoral immunodeficiency and the lack of IgA probably serve as the second pathogenic factor [19]. Secretory IgA and IgG antibodies act against periodontopathic and cariogenic consortia [17]. IgA deficiency promotes ulcerative periodontitis rather than a pustular vegetative disease of the oral mucosa, complicated by infection with *Elizabethkingia* spp. IgA-deficient patients show rapid and deep *Elizabethkingia* colonization during a preclinical phase followed by translocation to blood or, by inhalation, to the lower respiratory system [1], both of which are normally sterile sites.

CVID is a type of combined immunodeficiency [20], with the abnormal immune responses observed in these patients promoting bacterial infection primarily [21]. The clinical presence of pathergy, a condition in which a stimulus leaves an organism unduly susceptible, and the development of periodontitis at a site of minor trauma are manifestations of non-specific or non-selective inflammatory reactions. The CVID phenotype is characterized by abnormalities in the innate and adaptive immune responses. The third factor in pathogenesis in our patient was her very high IgM level and hyperviscosity, with rouleau formation that led to Raynaud phenomenon, microcirculation abnormality (microvasculopathy) with low oxy-reductive potential, ischemia-reperfusion and sometimes necrosis (Additional file 4: Figure. S3).

The mechanism of clinical pathergy observed in our patient was associated with activation of the classical complement cascade, as well as activation of the alternative complement pathway by pentameric IgM conglomerate or bacterial cell walls, resulting in the consumption of C4 and C3. Complement hyperactivation, its potential destruction of host tissues and its involvement in periodontal dysbiosis have been reviewed elsewhere [22]. High doses of IVIG prevent mucosal immune damage by scavenging complement fragments and binding anaphylatoxins to F(ab)_2_ [23].

This case description has several limitations, including the lack of a complete history of the patient’s treatment with antibiotics and steroids, as well as the unknown time of colonization. In contrast, the destruction of a fragile interspecific competition in the mucosal ecosystem may be caused by tripartite (host-pathogen, pathogen-drug, and host-drug) interactions, leading to periodontitis, post-antibiotic dysbiosis, post-steroids ulcers and immunomodulatory properties of IVIG (Additional file 4: Figure. S3). Early diagnosis and high-dose intravenous immunoglobulin therapy can restore periodontal symbiosis, reconstitute the humoral immune system, inhibit complement activation, and promote oral healing. Although the major adaptive host response is the production of IgA antibodies directed towards oral microbiota [19], recent findings show the crucial role of IgG, but not IgA or IgM [24]. The relationship between IgG titers and the severity of periodontitis has been described [25]. The clinical characteristics of our patient resemble those of *E. miricola* infection first reported in patients with stage IV mantle cell lymphoma after allogeneic stem cell transplantation with fluid overload and pulmonary hemorrhage [1]. Although *E. miricola* was present in respiratory and blood cultures, its epidemiology could not be determined. To our knowledge, this report describes the first case of opportunistic and oral infection (periodontitis, non-sterile site), humoral predisposing factors and immunomodulatory IVIG therapy characterized by *E. miricola* superinfection.

*E. miricola* should be considered a possible cause of infection (latent or apparent) in the oral cavity, spread by translocation or inhalation, and leading to severe pulmonary and bloodstream superinfection [1, 2, 11, 12] in patients with a late diagnosis of CVID, periodontitis and previous multi-antibiotic treatment. *E. miricola* is isolated from sterile sites during the late phase of the infectious process. However, this phase is preceded by the infection of susceptible body tissues and multiplication of the pathogen at the portal of entry, combined with the reaction of host tissues.

Autoinflammatory or granulomatous disease, and in some cases pathergy, observed in CVID co-exist with ubiquitous interactions of pathogens and non-immunocompetent, IgG/IgA-deficient hosts. Such interactions are the hallmark of CVID. The therapeutic regimen should be adapted to existing clinical and microbiological conditions. Early assessment of IgG, IgM and IgA concentrations, early identification of the pathogens by MALDI-TOF mass spectrometry, preemptive levofloxacin therapy and immune reconstitution by IVIG should protect against the severe, sometimes fatal, outcomes of *E. miricola* infection. MALDI-TOF mass spectrometry may become a method of choice for identification of *Elizabethkingia* infections.

## Additional files

Additional file 1: Figure S1. Case report timeline illustrating the natural history of the patient’s disease (CVID) and the course of the opportunistic *Elizabethkingia miricola* superinfection. (DOC 52 kb)

Additional file 2: Figure S2. Periodontitis caused by *Elizabethkingia miricola* in this patient. Gingival recession, resulting in apparent tooth lengthening. (DOCX 857 kb)

Additional file 3: Table S1. Laboratory data during admission and its significance. (DOCX 15 kb)

Additional file 4: Figure S3. Pathogenesis of and factors predisposing to opportunistic *Elizabethkingia miricola* superinfection in humoral immunodeficiency; i.e. common variable immunodeficiency. (DOCX 148 kb)

## Abbreviations

CVID: Common variable immunodeficiency
EM: *Elizabethkingia miricola*
ESID: European Society of Immune Deficiencies
MALDI-TOF: matrix-assisted laser desorption ionization—time of flight mass spectrometry
