# Supplementary material for: Elizabethkingia miricola as an opportunistic oral pathogen associated with superinfectious complications in humoral immunodeficiency: a case report
Source: BMC Infect Dis. 2017 Dec 12;17:763. doi: 10.1186/s12879-017-2886-7 (PMC5727958; doi:10.1186/s12879-017-2886-7)
Supplement: Supplementary file 1 — Case report timeline illustrating the natural history of the patient’s disease (CVID) and the course of the opportunistic Elizabethkingia miricola superinfection. (DOC 52 kb) [file 12879_2017_2886_MOESM1_ESM.doc]

| 3 year: first symptoms (sinopulmonary infections) | **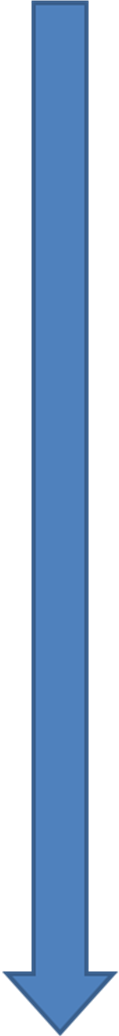** | Laboratory: non-responder after vaccination |
| --- | --- | --- |
| Childhood: recurrent pneumonia type A Influenza (despite vaccination) |  |  |
|  |  |  |
| Severe septicemia with *Enterobacteriaceae* | **14 Y.O.** | No hypogammaglobulinemia observed, lack of seroconversion to HBsAb |
| Episode of Raynaud phenomenon | **30 Y.O.** | Hypergammaglobulinemia |
| First episode of multiple ulcers in the oral cavity: topical therapy by dentist (without antibiotics) | **32Y.O.** |  |
| Oral mucositis, dry mouth   ↓  refractory periodontitis | **34 Y.O.** | Caphosol – temporary improvement   Nonsurgical cleaning below the gum line and steroid therapy ineffective. |
|  |  |  |
| Prolonged severe bronchopneumonia | **35 Y.O March** | Empirical antibacterial therapy with high dose amoxicillin (1000 mg t.i.d.) plus clavulanate (200 mg t.i.d.) prescribed by general practitioner |
|  |  | cefadroxil (500 mg b.i.d) |
| Hospitalization  Admission to the Department of Pulmonology:  Periodontitis exacerbation |  | ceftriaxone (1000 mg q24h) |
|  |  | azithromycin (500 mg q24h) |
| Admission to the Department of Immunology: Hypergammaglobulinemia with very high IgM, low IgG and complement consumption (cold agglutinin disease) CVID diagnosis | **. April** | crystalloids (rouleau formation reversible) and suboptimal body temperatures  Initial IVIG therapy (0.4 g/kg) every week |
| Rapidly progressing periodontitis |  |  |
| *Elizabethkingia miricola* in periodontal pocket | **May** | levofloxacin (750 mg/day for 21 days); IVIG to maintain IgG >500 mg/dl |
|  |  |  |
| Complete mucosal ulceration healing | **June** | Stable IgG level 700-900 mg/dl |

**Present**

**No episodes of periodontitis, Mild bronchopulmonary symptoms, IgM-** **paraproteinemia**

**Figure S1.** Case report timeline illustrating the natural history of the patient’s disease (CVID) and the course of the opportunistic *Elizabethkingia miricola* superinfection.
