# Supplementary material for: Elizabethkingia miricola as an opportunistic oral pathogen associated with superinfectious complications in humoral immunodeficiency: a case report
Source: BMC Infect Dis. 2017 Dec 12;17:763. doi: 10.1186/s12879-017-2886-7 (PMC5727958; doi:10.1186/s12879-017-2886-7)
Supplement: Supplementary file 2 — Periodontitis caused by Elizabethkingia miricola in this patient. Gingival recession, resulting in apparent tooth lengthening. (DOCX 857 kb) [file 12879_2017_2886_MOESM2_ESM.docx]

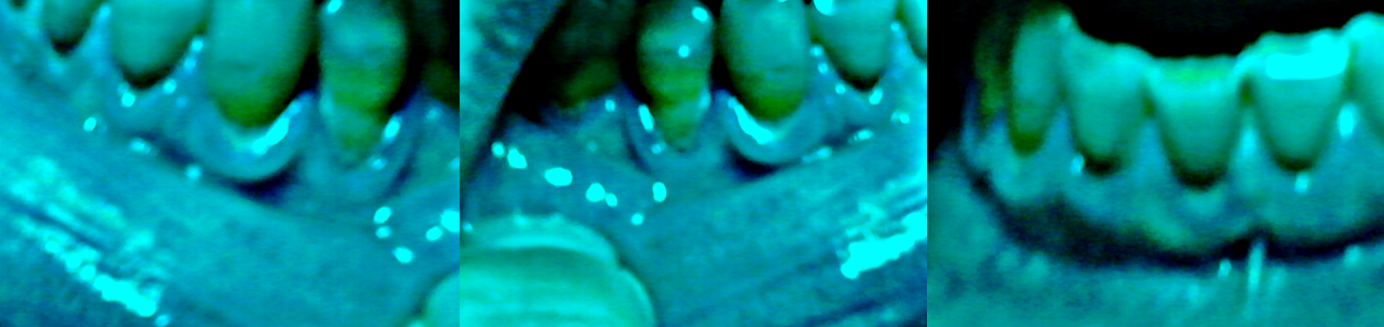


Additional file 2: Figure S2. Periodontitis caused by *Elizabethkingia miricola* in this patient. Gingival recession, resulting in apparent tooth lengthening.
