# Supplementary material for: Elizabethkingia miricola as an opportunistic oral pathogen associated with superinfectious complications in humoral immunodeficiency: a case report
Source: BMC Infect Dis. 2017 Dec 12;17:763. doi: 10.1186/s12879-017-2886-7 (PMC5727958; doi:10.1186/s12879-017-2886-7)
Supplement: Supplementary file 3 — Laboratory data during admission and its significance. (DOCX 15 kb) [file 12879_2017_2886_MOESM3_ESM.docx]

Additional file 3: Table S1. Laboratory data during admission and its significance.

| **Test** | **Results** | **Reference value and comments** |
| --- | --- | --- |
| AST [U/l] | 45 | 5-34 |
| ALT [U/l] | 66 | 0-55 |
| GGTP [U/l] | 77 | 9-36 |
| ALP [U/l] | 95 | 40-150 |
| Bilirubin [mg/dl] | 0.3 | 0.2-1.2 |
| Uric Acid^1^ [mg/dl] | 6.8 | 2.6-6 |
| Urea [mg/dl] | 23 | 15-40 |
| CRP | 5.4→6.5 | 0.2-5 |
| ESR ^2^ (Erythrocyte sedimentation rate) | 50→73 | 22 |
| Anitinuclear^3^ and other^4^ autoantibody | (-) | Autoimmunity -negative |
| IgG [mg/dl] | <50 | 700-1600 |
| IgM [mg/dl] | 1789 | 40-230 |
| IgA^5^ [mg/dl] | <1 | 70-400 |
| IgE^5^ [IU/ml] | <5 | 15-114 |
| C3 [mg/l] | 74.5 | 90-180 |
| C4 [mg/l] | 5.22 | 10-40 |
| Cold agglutinins | +++ | agglutination and rouleau formation, lack of IgG (warm) agglutinins |
| Lymphocyte level [/μl] | 1550 | 1000-4500 |

^1^ Uric acid is synthesized in liver and intestinal mucosa and is indicative to destruction of nucleoproteins e.g. in lymphoproliferative disease, multiple myeloma or hemolytic anemia.

^2^ Westergren formula: ♀ (age+10)/2 – ESR has several disadvantages that prevent it from being effective in monitoring acute inflammation or tissue injury. However, the ESR remains useful here for detection of paraproteinemia and microcirculatory monitoring, which do not necessarily provoke an acute phase response. Active paraproteinemia usually cause only a minor increase in CRP (in the range 1-6 mg%), although the ESR may be high in hyperviscosity with rouleau formation.

^3^ Immunoblot assay against: RNP, Sm, SS-A, SS-B, Ro-52, Scl-70 PM, Jo-1, Centromere B, PCNA, dsDNA, nucleosomes, histones.

^4^ Anti-mitochondrial, anti-liver-kidney microsome, rheumatoid factor class IgG and IgM.

^5^ IgG, IgA, IgE are below detection limit (nephelometry).
