# Supplementary material for: Elizabethkingia miricola as an opportunistic oral pathogen associated with superinfectious complications in humoral immunodeficiency: a case report
Source: BMC Infect Dis. 2017 Dec 12;17:763. doi: 10.1186/s12879-017-2886-7 (PMC5727958; doi:10.1186/s12879-017-2886-7)
Supplement: Supplementary file 4 — Pathogenesis of and factors predisposing to opportunistic Elizabethkingia miricola superinfection in humoral immunodeficiency; i.e. common variable immunodeficiency. (DOCX 148 kb) [file 12879_2017_2886_MOESM4_ESM.docx]

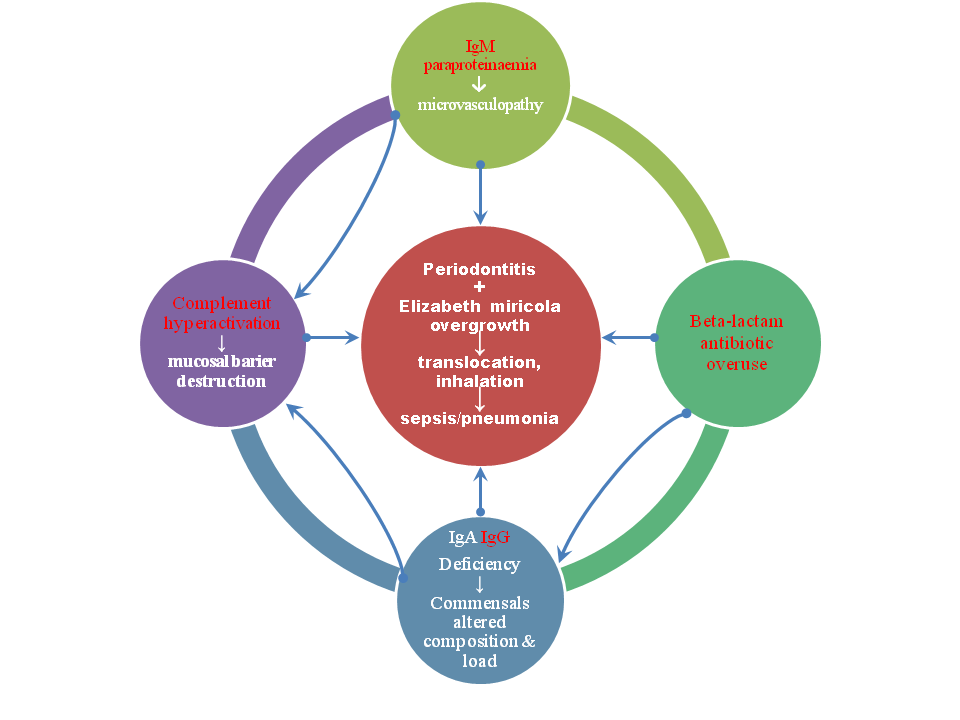


Additional file 4: Figure S3. Pathogenesis of and factors predisposing to opportunistic *Elizabethkingia miricola* superinfection in humoral immunodeficiency; i.e. common variable immunodeficiency.

Red font indicates proposed therapeutic strategy:

1. IgG replacement therapy that induce complement scavenging and specific response directed to oral microbiota
2. Early diagnosis with MALDI-TOF MS and beta-lactams withdrawal
3. Direct pharmacological complement inhibition (e.g. application of C1 inhibitor)
4. IgM paraproteinemia, cold agglutinins disease and rouleau formation that are reversible by saline dilution and suboptimal body temperatures.
